# Increased HIV in Greater Kinshasa Urban Health Zones: Democratic Republic of Congo (2017–2018)

**DOI:** 10.1186/s12981-020-00322-y

**Published:** 2020-11-23

**Authors:** Mohammad Pour, Linda James, Kamlendra Singh, Samuel Mampunza, Franklin Baer, JoAnna Scott, Michael G. Berg, Mary A. Rodgers, Gavin A. Cloherty, John Hackett Jr, Carole P. McArthur

**Affiliations:** 1 Pathology Department, Truman Medical Center, 2301 Holmes St, Kansas City, MO 64108 USA; 2 Department of Oral and Craniofacial Sciences, University of Missouri-Kansas City School of Dentistry, 650 E 25th Street, Kansas City, MO 64108 USA; 3 Université Protestante au Congo, Croisement de l’avenue de Libération et du Boulevard Triomphal, Kinshasa, Democratic Republic of Congo; 4 IMA World Health, 1730 M St NW Suite 1100, Washington, DC 20036 USA; 5 Department of Veterinary Pathobiology, University of Missouri, Columbia, MO 65211 USA; 6 Bond Life Sciences Center, University of Missouri, Columbia, MO 65211 USA; 7 Division of Clinical Microbiology, Department of Laboratory Medicine, Karolinska Institute, Stockholm, Sweden; 8 SANRU NGO, 76 Ave. de Justice, Kinshasa-Gombe, Democratic Republic of Congo; 9 Research and Graduate Programs, University of Missouri-Kansas City School of Dentistry, 650 E 25th Street, Kansas City, MO 64108 USA; 10 Abbott Laboratories, 100 Abbott Park Rd, Abbott Park, IL 60064 USA; 11 University of Missouri-Kansas City School of Medicine, 2301 Holmes, Street, Kansas City, MO 64108 USA

**Keywords:** HIV, Democratic Republic of the Congo, HIV prevalence

## Abstract

**Background:**

Diagnosis of people living with HIV (PLHIV) is the first step toward achieving the new Fast Track Strategy to end AIDS by 2030: 95-95-95. However, reaching PLHIV is especially difficult in resource-limited settings such as the Democratic Republic of Congo (DRC), where reliable prevalence data is lacking. This study evaluated the prevalence of HIV in patients in the urban Kinshasa area.

**Methods:**

Individuals seeking healthcare were tested for HIV between February 2017 and July 2018 at existing Kinshasa urban clinics. The study was conducted in two phases. Case finding was optimized in a pilot study phase using a modified cell phone-based Open\Data Kit (ODK) collection system. HIV prevalence was then determined from data obtained between March–July of 2018 from 8320 individuals over the age of 18 years receiving care at one of 47 clinics in Kinshasa.

**Results:**

The prevalence of HIV in our study was 11.0% (95% CI 10.3–11.6%) overall and 8.14% in the subset of N = 1240 participants who were healthy mothers seeking prenatal care. These results are in sharp contrast to President's Emergency Plan for AIDS Relief (PEPFAR) estimates of 2.86%, but are consistent with data from surrounding countries.

**Conclusion:**

While this data is sub-national and reflects an urban healthcare setting, given the large population of Kinshasa and rapidly changing age demographics, the results suggest that HIV prevalence in the DRC is substantially higher than previously reported.

## Introduction

The Democratic Republic of the Congo (DRC) consists of 26 provinces, with 15% of the total DRC population living in the largest province of Kinshasa. Kinshasa has a rapidly growing population of 11.3 million [1], more than 50% who are younger than 22 years of age, and few are older than 50 years. Characteristic of sub-Saharan Africa, this expanding demographic consists primarily of individuals in their sexually active years [2, 3]. Widespread poverty, decades of social or political unrest and epidemics, have led to the lack of a functional public health system in the DRC. Limited access to healthcare by most citizens is due to poor infrastructure, lack of resources, ineffective data collection, and administrative inefficiencies [4]. In 2007, the Demographic Health Surveys (DHS) estimated that only 9% of adults in the DRC knew their HIV status. This was due in part to the limited availability of HIV counseling and testing [4]. Furthermore, the DRC National AIDS Control Program estimates that only 10% of people living with HIV who are eligible for an anti-retroviral therapy program, are enrolled [4]. It is critical to identify HIV positive people and connect them with anti-retroviral therapy to reduce viral loads and prevent the spread of HIV. The U.S. President’s Emergency Plan for AIDS Relief (PEPFAR) is prioritizing population-based, HIV-focused household surveys, as a means for monitoring HIV incidence, prevalence, and viral load. Unfortunately, the current landscape in the DRC provides a major challenge to that goal and HIV epidemic control.

Fragmented reports from the DRC show that high quality and reliable data regarding the HIV status of citizens is difficult to obtain from this country. Sero-prevalence of HIV in the DRC determined from DHS has generally reported low rates of infection over many years [4–7]. Likewise, UNAIDS estimates that only 560,798 out of an estimated 74.6 million (0.75%) in the DRC live with HIV [8]. World Health Organization (WHO) data for DRC, based upon the 2012–2013 DHS statistics and UNAIDS 2015 Spectrum *estimates* (version 5.51) [4], reports HIV prevalence in individuals between ages 15–49 to be 1.2%, with a range between 0.6 and 1.7%. According to the World Bank, the average peak prevalence of 2.2% for adults (15–49) occurred between 1996 and 2000 and has been steadily declining until the present. However, in 2016, President’s Emergency Plan for AIDS Relief (PEPFAR) tested 915,609 individuals and showed a higher overall prevalence of 2.86%. A similar study in 2017 showed a prevalence of 2.93% [3, 4]. DHS data have shown consistently that HIV is more prevalent in urban than in rural areas and higher in women than men in the DRC. In this report, we conduct an extensive study focusing on widely distributed clinical sites throughout the city of Kinshasa and find a much higher HIV prevalence of 11%. Despite the inherent bias of selecting people who seek healthcare, given the large population of Kinshasa, the much greater estimates in surrounding countries [4, 9, 10], the 8% prevalence in healthy pregnant women, and the fidelity with which this data was collected, the overall prevalence of HIV in the DRC should be re-evaluated.

## Methods

### Study population

This was an observational study with data collected from February 2017 through July 2018 from 47 clinical sites in 24 health zones in Kinshasa within an approximate 154 square mile (400 km^2^) area. Participants (primarily adults) received a rapid HIV test at one of 35 hospitals or clinics in 2017 during the pilot phase, and 47 similar sites (12 additional) in 2018. The sites were in different urban health zones and represented a range of available levels of healthcare delivery. Patient’s blood was drawn in an EDTA tube at the collection sites, centrifuged to prepare plasma, and transferred to ice within 2 h to our laboratory at Université Protestante au Congo (UPC). Plasma specimens were tested by HIV serology and confirmed following the National and WHO recommended algorithm at the sites. Multiple rapid tests were used as follows (Determine HIV-1/2 [Determine; Alere, USA], Uni-Gold HIV [Uni-Gold; Trinity Biotech, Ireland], and Vikia HIV 1/2 [Vikia; bioMérieux, France]). All rapid tests have > 99–100% sensitivity and specificity for the detection of HIV-1 and HIV-2.

### Case finding optimization

Enhanced efficiency and turn-around time were achieved by modification of a cell phone-based Open\Data Kit (ODK) [11]. The ODK is a non-texting based application that transfers simple data and GPS coordinates directly to the cloud for subsequent downloading to our server at UPC. Barcoded cell phone data upload was piloted from January through February 2018, and implemented in March–July 2018 for prevalence calculations. Pilot testing was initiated at three sites and rapidly expanded to a network of 47 productive sites over the 18-month period. Numbers of positive and negative HIV tests were calculated each month across all clinics with the goal of locating and continuing testing at sites with a cut-off of two or more HIV positive patients detected per month. This was to ensure we were utilizing resources and directing personnel to areas with the most need. Eleven sites did not meet the criterion for continuation; four sites were dropped in 2017 and seven in 2018. Before the study began, a team of five physicians, a technician, and two faculty members carried out HIV testing and quality control training to improve case finding. Training continued throughout the study and the process was refined in an ad hoc manner, with each physician managing an average of eight sites, all centrally coordinated at UPC.

### Statistical analysis

Participants missing HIV results, age or gender were excluded from the analysis. HIV prevalence was calculated overall, as well as by month and year for each phase of the study. The reported prevalence was restricted to data collected from March through July of 2018 during the implementation phase. The prevalence overall was calculated by gender and age categories. Differences in prevalence by age (continuous) was calculated using a two-sample t-test with unequal variances. Differences in prevalence by age categories, by gender, and by age and gender together were evaluated using a Chi-square test or a two-sample test of proportion. The significance level was set to 0.05. All analyses were performed using Stata 15.1 (StataCorp. Stata Statistical Software: Release 15.1. College Station, TX, USA: StataCorp LLC, 2017).

## Results

**Fig. 1:**
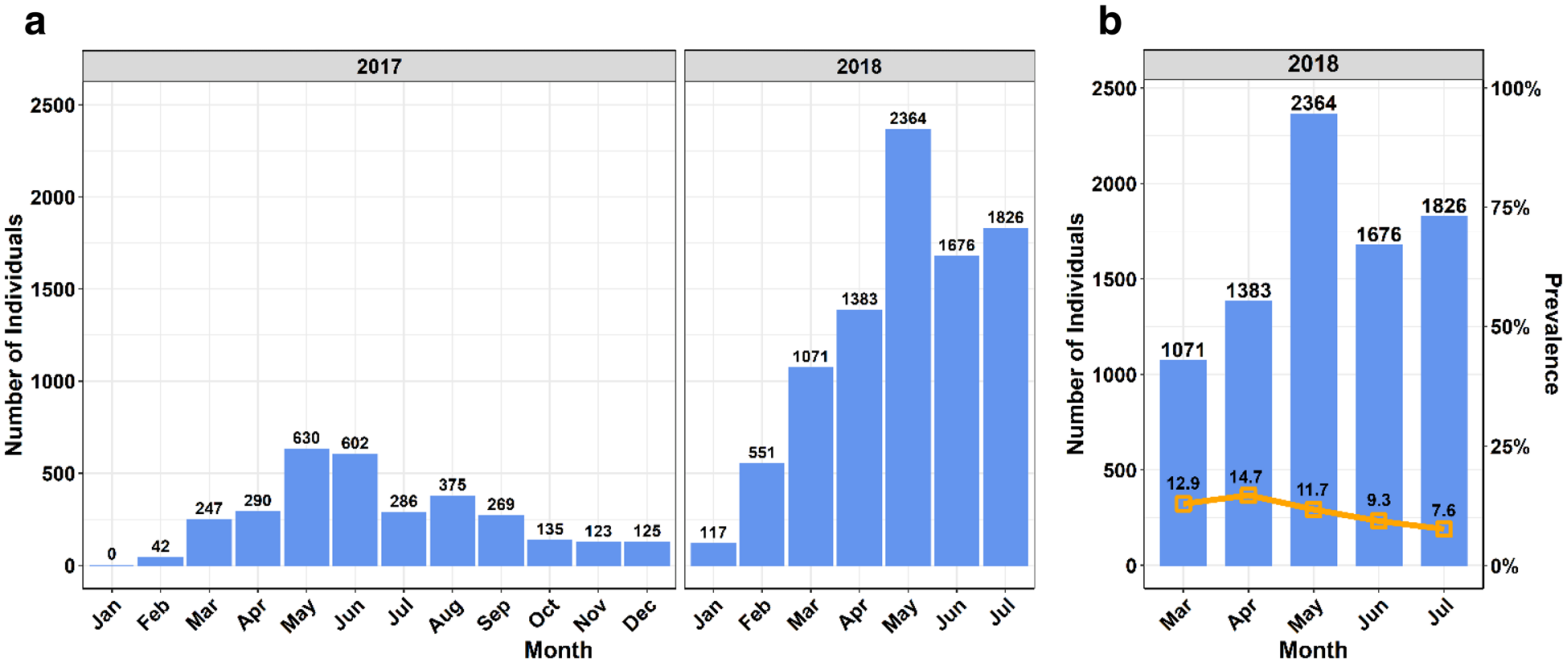
Number of individuals tested and prevalence of HIV positive individuals. **a** Shows the number of individuals tested at 35–47 sites in the Kinshasa network during pilot phase (2017), and implementation phase (2018). The testing occurred from February 2017 through July 2018. **b** Shows the number of individuals tested, and prevalence of HIV positive individuals during the period of analysis. HIV prevalence (%) at 47 network sites in Kinshasa from March–July 2018 using the ODK data collection modification, is depicted by orange squares and connected through orange line in secondary Y-axis. All figures were generated using statistical program R [18], and graphical user interface (GUI) RStudio [19]

The goal of this cross-sectional study was to determine the prevalence of HIV infections in a diverse urban cohort in the DRC capital city of Kinshasa, and to evaluate a new data collection tool. During the pilot phase of the study (January 2017 through February 2018), staff were trained and the number of sites included in the study was expanded from 3 to 44. The numbers screened per month ranged from only 42–630 patients within this period (Fig. 1a). A cell phone-based Open\Data Kit (ODK) adopted during the implementation phase greatly improved the accuracy and reliability of data collection compared to recording by hand. After incorporation of improved and faster data collection procedures and the utilization of the ODK technology in March 2018, there was a large increase in the number of individuals screened (1071–2364 participants/month). Subsequently, the estimated prevalence stabilized (Fig. 1b).

During the implementation phase from March to July of 2018, there were 8465 individuals 18 years or older that were tested for HIV at 44 clinics (Fig. 1B). Of those, 8320 (98.3%) had valid sex and HIV serology data. A total of 912 participants tested positive, and a prevalence of 11% (range of 7.6–14.7%) was obtained. The 8320 individuals with valid data were on average 34.9 (± 12.5) years old. The majority were female (67.9%), in the age ranges 25–44 (60.3%), and had an HIV prevalence of 11.0% (95% CI 10.3–11.6%) (Table 1). Individuals who tested positive for HIV were significantly older than those who tested negative (39.0 vs. 34.4 years, p < 0.001). Additionally, HIV prevalence was significantly higher in the 45–54 year old age group (23.6%) and lower in the 65 years or older age group (3.9%) (p < 0.001) regardless of gender (Table 1). Likewise, the overall prevalence of HIV was not significantly different by gender, with overall female prevalence at 10.5% and male prevalence at 11.9% (p = 0.069).

**Table 1:** HIV prevalence by age groups and gender

	Entire sample	HIV prevalence			p-value*
		Overall (N = 8320)	Female (N = 5652)	Male (N = 2668)	
	N (%)	100%	68%	32%	
Age (years)					
18–24	1687 (20.3%)	4.7%	5.0%	4.0%	0.399
25–34	3144 (37.8%)	8.3%	8.5%	7.9%	0.636
35–44	1877 (22.6%)	15.8%	15.8%	15.8%	0.985
45–54	844 (10.1%)	23.6%	23.1%	24.3%	0.686
55–64	514 (6.2%)	12.7%	12.8%	12.4%	0.894
65+	254 (3.1%)	3.9%	2.7%	5.7%	0.232
Total	8320 (100.0%)	11.0%	10.5%	11.9%	0.069
p-value**		< 0.001	< 0.001	< 0.001	

*Calculated using a 2 sample test of proportion

**Calculated using a Chi-square test

To evaluate whether the high rate of HIV in our study was due to a sampling bias towards unhealthy individuals seeking healthcare, the data were parsed to focus only on healthy, expecting mothers attending prenatal clinics. Over the same 5 months (March 2018–July 2018), a total of 1240 pregnant women were screened at 8 different facilities, and 101 of them were HIV positive. The percentage of monthly positives consistently ranged from 7.1 to 11.7%. The combined prevalence of 8.14% for pregnant women, while lower than the overall observed in this study, is still far above recent national estimates of 2.9% [3, 4].

## Discussion

**Fig. 2:**
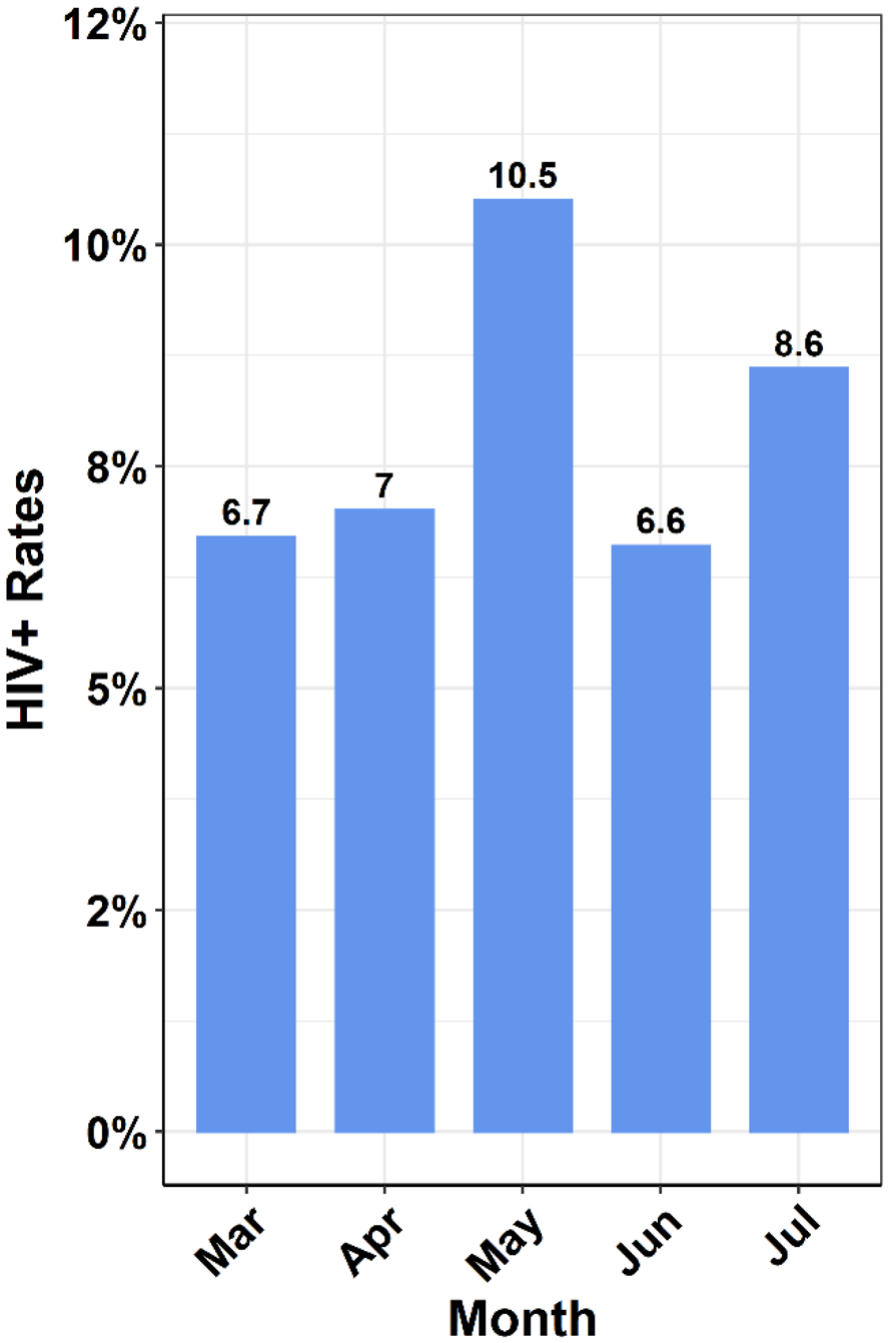
Prevalence of HIV in generally healthy women attending maternity clinics for prenatal care during the months of March 2018 to July 2018

In this study, we developed and sustained an efficient HIV screening network spanning 18 months (February 2017–July 2018). Our results from a large cohort of people seeking healthcare (N = 8320) reveals a much higher HIV rate of 11.0% in Kinshasa than national reports of 1.0–3.0% [1, 4, 6, 10, 12]. We expected to find a higher prevalence of HIV among people seeking healthcare compared to the general population, but the magnitude of the difference observed was unexpected. It is pertinent to note, however, the generally higher HIV prevalence reported from several countries surrounding the DRC [10]. These include the Republic of the Congo [4] and Zambia that has wide-ranging prevalence reports within Zambia of up to 30% [9]. Secondly, the prevalence of HIV among healthy mothers (Fig. 2) seeking prenatal care or post-natal care for their infants was also much higher (8%) than previously reported. This strengthens our hypothesis that HIV prevalence is much higher in the DRC than previous estimates in the general population. It is not possible to compare sampling methods in the above countries from reports but sampling methodology is a likely source of the wide-ranging prevalence reports in Zambia, for example. It is noteworthy that the DRC and Cote d’Ivoire data (Ref. [4] panel D, p7) show total HIV deaths exceed new HIV cases, indicating a loss of epidemic control. A confounding factor could result from individuals who tested twice under different names, but we were not able to verify that this occurred. Indeed, the ODK bar-coded system of uploading data reduces the potential for this to occur. It is also possible the phenomenon of spatial variability impacted our results. This occurs when clusters of high transmission are obscured by low national prevalence estimates. This could possibly explain the differences between our data and previous prevalence studies [13]. PEPFAR studies and a few anecdotal reports suggest an increased incidence at a few sites in Kinshasa. However, legitimate, well-controlled incidence studies have not been carried out in the DRC for decades suggesting these are urgently needed. Furthermore, it is important to note that most of the previous WHO, UNAIDS, World Bank and Global Fund reports are only *estimates* based upon modeling of earlier data from limited National surveys carried out as early as 2011. Years of political and social unrest have resulted in a poorly supported public health system that challenge reliable data acquisition by international agencies in the DRC and other low resource environments. It is widely reported that an increase in AIDS-related deaths has occurred recently in many Central African countries further suggesting DRC’s situation is much bleaker than envisaged [10]. The accuracy of national estimates is critical for programmatic planning, effective scale-up, and cost-effective use of resources to obtain epidemic control.

Factors responsible for successful case-finding in our study included personnel availability and training, technical and diagnostic resources, physician diagnostic practices, and location. We determined that limitations in data recording were often the result of under-compensated personnel, personnel absences, poor supervision, and insufficient training. To mitigate this, our supervisors were well-trained, paid (often personnel are volunteers), and resided at the same clinical sites throughout the entire study. During the site selection process, we targeted maternity clinics where women were encouraged to determine their HIV status during pregnancy, as well as infectious disease and STD clinics that attract patients seeking healthcare. Specific clinical sites exhibited an increased prevalence of HIV, generally associated with large hospitals (with infectious disease clinics), and in densely populated areas of Kinshasa. Our results reflect conclusions from the 2017 PEPFAR report [4] and the Global Burden of Disease (GBD) study [14] that not surprisingly show some centers have higher numbers of HIV patients than others [4, 13].

It is important to note that HIV/AIDS is a heterosexual disease throughout Africa and most of the developing world [15]. In the US, Europe and the developed world, HIV is most associated with men who have sex with men (MSM) and with intravenous drug abusers (IVDA). This leads to a significantly different distribution of HIV within the population in Africa at large, compared to Westernized countries. While our study showed no differences between male and female rates of infection, we estimate that women account for 58% of the people living with HIV (PLHIV) in this sub-Saharan region. This skewed distribution has existed for years with respect to women, who on average acquire HIV as much as 5–7 years earlier than their male peers [15]. In our experience in sub-Saharan Africa, women are more generally open to HIV testing than African men. This is possibly because women interact with available health services more frequently. They are responsible for child-care and receive HIV counseling and education during pregnancy testing, prenatal care and delivery. There are some mother-to-child transmission (MTCT) programs but these are under-funded and poorly resourced, challenging patient follow-up. For example, it is often very difficult to obtain confirmation of infant HIV status by PCR. Our study population was comprised of 68% female participants, which reflects the trend (Table 1). With a young, sexually active, and rapidly expanding population in DRC, it is imperative that women in particular, are tested and informed of their status to help reverse this troubling trend.

## Conclusions

The goal of PEPFAR/Global Fund is to achieve the UNAIDS 90–90–90 treatment target by 2030 [16]. Thus, 90% of people living with HIV should know their HIV status, 90% of people who know their HIV-positive status are accessing treatment, and 90% of people on treatment will have suppressed viral loads. Antiretroviral therapy for North Africa including the DRC lags far behind other regions although coverage has increased from 2010 to 2015 (Sources: GARPR 2016; UNAIDS 2016 *estimates*). The above UNAIDS goals are exceedingly ambitious for the DRC given the general decline in services [17] and the results of this study. There has been unmitigated political unrest over the past 5–6 years, the recurrence of Ebola and unmet funding provided by the DRC Government. Only 9% of adults in the DRC know their HIV status [4] and only 10% of HIV patients receive cART [8]. A concerted international effort is critical for global security, to meet the 90-90-90 goals and to have any possibility of ending AIDS by 2030.

## Data Availability

Raw serologic data will be available on the University of Missouri-Kansas City school of Dentistry web site.

## Abbreviations

cART: Combination antiretroviral therapy
DHS: Demographic Health Surveys
DRC: Democratic Republic of Congo
GBD: Global burden of disease
GPS: Global Positioning System
IVDA: Intravenous drug abusers
MSM: Men who have sex with men
ODK: Open\Data Kit
PEPFAR: President's emergency plan for AIDS relief
PLHIV: People living with HIV
STD: Sexually transmitted disease
UNAIDS: Joint United Nations Programme on HIV/AIDS
